
JOURNAL INFORMATION
==============================
NLM Title Abbreviation: ScientificWorldJournal
Journal ID: ScientificWorldJournal
NLM Journal ID: 101131163
Journal ID: TSWJ

The Scientific World Journal

ISSN: 2356-6140
EISSN: 1537-744X
Publisher: Wiley

ARTICLE INFORMATION
==============================
PMCID: PMC6084199
PMID: 30147539
DOI: 10.1100/tsw.2001.250
Article ID: 920576
Article version: 1
Subjects: Short Report

Chronic Alcohol Consumption Attenuates Apoptosis by Down Regulation of P53 Expression in the Rat Pancreas

Fortunato Franco *
Gates Jr. Lawrence K.
Division of Digestive Diseases and Nutrition, Department of Internal Medicine, University of Kentucky, Lexington, KY 40536, USA
*Franco Fortunato: ffort0@pop.uky.edu
Publication date: 2001
Electronic publication date: 2001 Apr 4
Volume: 1
First page: 68
Last page: 68
Copyright: Copyright © 2001 Franco Fortunato and Lawrence K. Gates Jr..
Copyright year: 2001
License: This is an open access article distributed under the Creative Commons Attribution License, which permits unrestricted use, distribution, and reproduction in any medium, provided the original work is properly cited.
License URL: https://creativecommons.org/licenses/by/3.0/

==============================
Miami *Nature Biotechnology* Short Reports
*TheScientificWorld* (2001) 1 (S3), 68SR
ISSN 1532-2246; DOI 10.1100/tsw.2001.250

### CHRONIC ALCOHOL CONSUMPTION ATTENUATES APOPTOSIS BY DOWN REGULATION OF P53 EXPRESSION IN THE RAT PANCREAS

Franco Fortunato* and Lawrence K. Gates Jr.

Division of Digestive Diseases and Nutrition, Department of Internal Medicine,
University of Kentucky, Lexington, KY 40536
* ffort0@pop.uky.edu

**INTRODUCTION.** Heavy alcohol intake is a major cause of pancreatitis in humans, possibly through the regulation of apoptosis. Dysregulation of apoptosis has been considered a causative factor in several diseases. Emerging data show that ethanol intoxication interferes with the rate of apoptosis, which is deleterious to several organs, including the pancreas (1). We investigated a possible mechanism by which chronic alcohol feeding in rats may alter pancreatic homeostasis through a reduced apoptotic rate.

**METHODS.** Rats were fed a liquid diet (Lieber-DeCarli) containing either ethanol or maltose dextrin in isocaloric amounts for 14 weeks. Pancreatic injury was assessed by plasma a-amylase activity. Apoptosis was detected by *in situ* TUNEL assay, Caspase-3 and Caspase-8 activity. Messenger RNA expression of apoptotic factors and cytokines was investigated by RNase protection assay, while cpp32 (caspase-3), Fas ligand and PAP-1 mRNA expression was determined by semi-competitive RT-PCR. p53, Fas ligand, Bax and Bcl-2 protein expression was analyzed by western blotting.

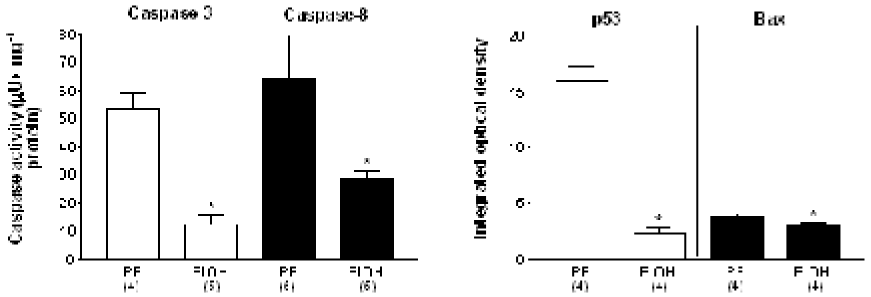

**RESULTS.** Chronic alcohol feeding significantly reduced serum a-amylase activity. p53 protein expression was dramatically diminished by alcohol administration. Caspase-3 mRNA and enzyme activity, caspase-8 activity, Bax mRNA and protein as well caspase-1 and -2 mRNA expression were also significantly reduced. Pancreatitis associated protein (PAP), a marker of pancreatic inflammation, showed a significantly reduced mRNA expression. We also found a non-significant trend toward decreases TUNEL positive nuclei in pancreata of alcohol fed rats. Chronic alcohol feeding suppressed the rate of apoptosis in the pancreas by diminishing the caspase activity and reducing the mRNA transcription level of intracellular pro-apoptotic factors. Alcohol did not affect the mRNA expression or the protein level of either anti-apoptotic factors or the Fas/FasL system.

**DISCUSSION.** Alcohol or its intermediates may directly interfere with the mRNA transcription level of pro-apoptotic factors and PAP-1. These data suggest that chronic alcohol intake reduces the basal apoptotic rate, thus preventing the elimination of senescent acinar cells in the pancreas. These aged acinar cells, which would normally be eliminated by apoptosis, survive because of the reduced rate of apoptosis. It has been shown that exposure to alcohol reduces NF B activation (2). NF B seems to play an important role in down-regulation of p53, Bax and caspase expression, deceasing the expression of these pro-apoptotic factors. Thus, the senescent cells may develop a vulnerability to additional insults ($2^{nd}$ hits) such as bacterial endotoxin or neoplastic transformation, which may be responsible for pancreatitis or a predisposition for the development of pancreatic cancer.

**REFERENCES.**

1. Fortunato, F. and Gates, L. (2000) Pancreas 21(2), 174-180
2. Pandol, S.J., Periskic, S., Gukovsky, I., Zaninovic, V., Jung, Y., Zong, Y., Solomon, T.E., Gukovskaya, A.S., and Tsukamoto, H. (1999) Gastroenterology 117, 706-716
